# A streamlined procedure for advancing the detection and isolation of *Listeria monocytogenes* from artificially contaminated ground beef in a single working day

**DOI:** 10.1128/spectrum.01577-24

**Published:** 2025-02-25

**Authors:** Min Lin, Hanhong Dan, Jiewen Guan

**Affiliations:** 1 Canadian Food Inspection Agency, Ottawa Laboratory Fallowfield, Ottawa, Ontario, Canada; 2 Department of Biochemistry, Microbiology and Immunology, University of Ottawa, Ottawa, Ontario, Canada; Institut National de Santé Publique du Québec, Sainte-Anne-de-Bellevue, Québec, Canada

**Keywords:** *Listeria monocytogenes*, foodborne pathogen, culture enrichment, filtration, bacterial capture, detection and isolation

## Abstract

*Listeria monocytogenes*, a rod-shaped Gram-positive bacterium widely distributed in nature, can contaminate foods and represents a foodborne pathogen of public health significance causing a high mortality rate of 20%–30%. Rapid and reliable identification of foods and food-processing environments contaminated with *L. monocytogenes* is a crucial step in implementing effective intervention strategies to ensure food safety and limit the transmission of bacteria to humans. This study designed and refined a practical workflow to streamline and accelerate the detection of a low level of *L. monocytogenes* present in ground beef. The workflow coupled an abbreviated 5 h culture enrichment in PALCAM liquid medium with physical separation (filtration and centrifugation) to preprocess enrichment samples. Specific capture was achieved using magnetic separation with a bacteriophage endolysin-derived cell wall-binding domain in a Hyglos *Listeria* capture kit. Molecular detection was performed using a MicroSEQ *L. monocytogenes* RTi-PCR detection kit combined with a nested PCR strategy. Preprocessing of enrichment culture samples using a multi-stage filtration system constructed for the study or commercially available BagFilter Pull-up filter bags, in conjunction with centrifugation, enabled the recovery of ~30 colony-forming units (CFUs) from the enrichment culture of a 25 g ground beef sample artificially contaminated with 1 CFU of *L. monocytogenes*. Integration of magnetic separation into the workflow for capturing *L. monocytogenes* cells specifically from preprocessed samples and further cleaning up the samples yielded bacterial counts similar to those obtained by direct plating of preprocessed samples. The RTi-PCR-based molecular detection method integrated into the workflow was capable of detecting pure cultures of *L. monocytogenes* as low as 12.5 CFUs. Evaluation of the workflow using artificially ground beef demonstrated the consistent detection of *L. monocytogenes* within an 8 h workday in a 25 g sample unit containing the cell count as low as 2 CFU following a 5 h culture enrichment.

**IMPORTANCE:**

Consuming foods contaminated with the bacterial pathogen *Listeria monocytogenes* can lead to the development of human listeriosis, a severe and life-threatening foodborne illness. Timely detection of *L. monocytogenes* present at a low level in foods and food processing environments is a necessary measure to prevent the spread of the *Listeria*-associated illness. This study designed and evaluated a multi-step workflow for testing *L. monocytogenes* in artificially contaminated food samples. The workflow was composed of a short 5 h culture enrichment, filtration-based sample preprocessing, magnetic separation, a single-tube nested RTi-PCR, and culture plating. It allowed *L. monocytogenes* to be detected within 8 h from a 25 g ground beef sample containing the target cells as low as 2 colony-forming units, significantly improving and streamlining the detection methods for this important foodborne pathogen.

## INTRODUCTION

The Gram-positive bacterium *Listeria monocytogenes* is a facultative intracellular pathogen that can cause a serious and life-threatening human infection (referred to as listeriosis) upon consumption of contaminated foods in susceptible individuals, particularly pregnant women, newborns, older adults, and immunocompromised people (1). This pathogenic microorganism is one of the 17 species currently recognized in the *Listeria* genus, including nine *Listeria* spp. newly reported since 2009 (2). *L. monocytogenes* and *Listeria ivanovii* are the only two pathogenic species in the genus. *L. monocytogenes* infects humans and animals, while *L. ivanovii* predominantly infects ruminants and is rarely associated with human listeriosis (3). *L. monocytogenes* represents a foodborne pathogen of public health significance. Foodborne illness due to *L. monocytogenes* presents a high mortality rate of 20%–30% (4, 5) and is the third leading cause of death due to foodborne microbial infections in the United States (6). Strains of *L. monocytogenes* are genetically diverse and are serologically differentiated into at least 13 serotypes and phylogenetically grouped into four distinct lineages (I, II, III, and IV) (7). Although serotypes 1/2a, 1/2b, and 4b account for at least 95% of human listeriosis cases (8), the development of the laboratory capacity to detect strains of all *L. monocytogenes* serotypes in foods or other sample sources is highly desirable.

It is impossible to completely eliminate *L. monocytogenes* from foods or food-processing plants because the bacterium has the ability to persist in various food-associated environments (9), to form biofilms in food-contact surfaces (10), to grow at refrigerator temperatures, and to survive harsh environmental conditions such as freezing, high salts, and low pH (11–13). Rapid and accurate identification of foods and food-processing environments contaminated with *L. monocytogenes* is a crucial step for implementing effective intervention strategies to ensure food safety and control bacterial transmission from the sources to humans. This task, however, remains a serious challenge, given that the number of viable *L. monocytogenes* cells is often found low in food or environmental samples taken for laboratory tests, and thus, selective culture enrichment for a lengthy period of at least 48 h (i.e., primary enrichment for 18–24 h and likely secondary enrichment for 18–24 h) (14) is necessary to raise the bacterial count to a detectable level. Another hurdle is the presence of background microflora and various compounds in food matrices, leaving molecular detection assays such as PCR-based tests prone to inhibitory effects (15). It may be possible to reduce the turnaround time significantly for detecting *L. monocytogenes* in food or environmental specimens by designing an innovative testing strategy to streamline the process of removing food particles from culture-enriched samples, separation of target bacteria to remove background microflora and potential PCR inhibitors, specific detection by molecular methods, and confirmation of bacterial identity by traditional culture-based testing.

Detection of *L. monocytogenes* in food or environmental samples is a time-consuming and tedious process that may involve the use of multiple methods. Steps of the detection process include conventional microbiological techniques following selective culture enrichment (14, 16), concentration of bacterial cells using physical methods such as centrifugation and filtration (17, 18), separation of bacterial cells using magnetic beads pre-coupled with antibodies (19–26) or endolysin-derived cell wall-binding domains (CWBD) (27–29), and downstream detection using a wide range of tools such as immunological and molecular techniques (30). By employing a combination of culture enrichment, filtration, and centrifugation, immunomagnetic separation (IMS), and PCR in a system, a few studies demonstrated the feasibility of detecting *L. monocytogenes* in 24 h from food samples artificially contaminated with a low level of the pathogen (21). Further decrease in the test turnaround time for food or environmental samples contaminated with a low level of *L. monocytogenes* can be beneficial in multiple aspects as it enables a timely response for pathogen control, cuts unnecessary costs in laboratory resources, and prevents economic losses for food industries and healthcare burden due to the risk of foodborne listeriosis outbreak.

This study reports the design and evaluation of a test process of analyzing foods contaminated with 1–5 colony-forming units (CFU) of *L. monocytogenes* in 25 g food samples for the presence of target cells within a normal 8 h workday. *L. monocytogenes* contamination of fresh meats including ground beef was documented in published articles (31). Ground beef was thus used here to prepare artificially contaminated samples with *L. monocytogenes* serotype 4b strain LI0521 as a model organism for testing. By using culture enrichment for 4–5 h, a multi-stage filtration system (MSFS) constructed here or commercial filter bags together with centrifugation for removal of large food particles, magnetic beads covalently pre-conjugated with a bacteriophage endolysin-derived CWBD for bacterial separation and concentration, and real-time quantitative PCR (qPCR) for the downstream detection, we showed an improved, streamlined test process that enables the detection of *L. monocytogenes* in contaminated food samples within 8 h.

## MATERIALS AND METHODS

### Culture of *L. monocytogenes* for experimental use

*L. monocytogenes* LI0521 serotype 4b was used in the study. Bacterial cells were cultured from a single colony grown on a brain heart infusion (BHI; BD Diagnostic Systems, Sparks, MD) agar plate in 5 mL of BHI or PALCAM broth (with no antibiotics added; EMD Millipore, Darmstadt, Germany) at 37°C overnight with gentle shaking at 250 rpm. The optical density (OD) at 620 nm was determined for the overnight broth culture and used to estimate the cell count with an OD_620_ of 1 equivalent to 1 × 10^9^ CFU/mL (32). The cell concentrations were verified by plating serially diluted culture samples in phosphate-buffered saline (PBS) on BHI agar media and then counting the colonies after incubation at 37°C for 24 h.

### Design and construction of an MSFS

**Fig 1:**
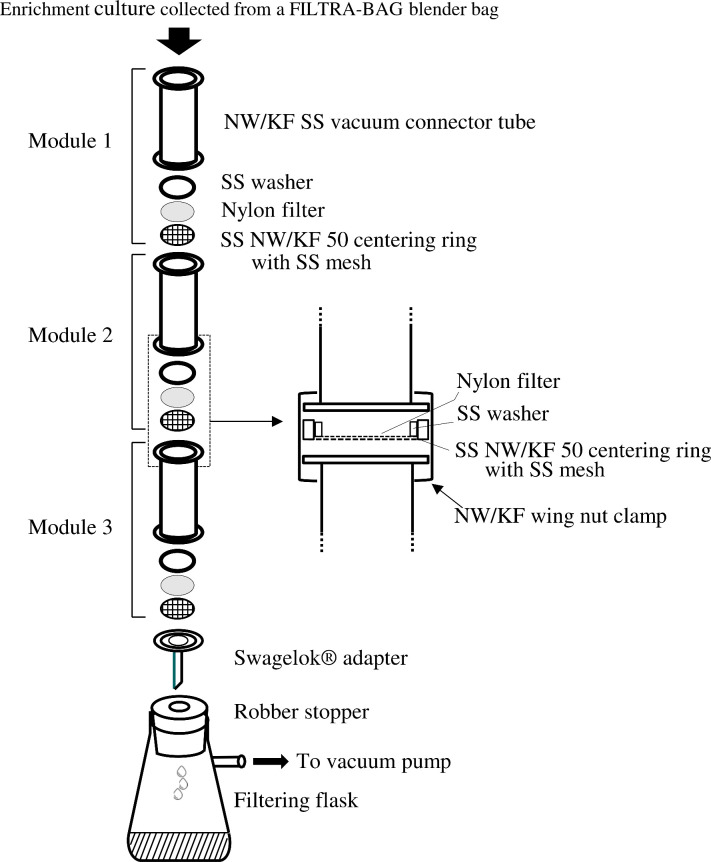
Schematic illustration of an MSFS. The system is constructed with an assembly of three series interconnected modules. Module 1 accepts an enriched culture sample input, while Module 3 connects to a filtering flask used to collect filtered culture samples.

An MSFS was constructed to harvest bacteria from food samples artificially contaminated with *L. monocytogenes* (Fig. 1). The system consists of three connecting filtration modules mounted vertically to a support stand with three 2-prong adjustable clamps (ThermoFisher, Ottawa, Ontario). Each module has an NW/KF SS vacuum connector tube (160 × 50 mm), an SS NW/KF 50 centering ring with an outer rubber Viton O-ring, a 60 mesh (250 microns) SS screen (Ted Pella Inc. CA), and an NY1H Nylon filter disc (47 mm) of desired pore sizes (180–40 microns; Millipore, Etobicoke. Ontario). To secure the Nylon filter in place during vacuum filtration, a specially designed SS washer (1.874 ± 0.005 OD; 1.559 ± 0.0051 ID; 0.093 ± 0.006 T; Boker’s, Minneapolis, MN) was placed on the edge of the filter disc laid on the mesh screen. The end-head assembly of connecting modules was braced and fastened using an NW/KF wing nut clamp (Ted Pella Inc., Redding, CA). This design allows for replacing filter discs with ease. The bottom module is coupled to a Swagelok adapter (Ted Pella, Inc. CA) with its outlet tube inserted into a filtering flask connecting to a KNF vacuum pump (ThermoFisher, Ottawa, Ontario).

### Preparation and culture enrichment of artificially contaminated ground beef samples

Ground beef was purchased from local supermarkets and tested for *L. monocytogenes* using the MFHPB-07 method (14). Samples (25 g each) of *Listeria* negative beef were placed in sterile 50 mL centrifuge tubes and stored at −20°C until use. Prior to use, beef samples were thawed at 4°C overnight followed by inoculation each with 1 mL of sterile PBS (pH 7.4) containing 0, 1, 2, 5, 50, and 200 CFUs of *L. monocytogenes* strain LI0521 (serotype 4b). Both contaminated and non-contaminated samples were incubated at 4°C for 20–24 h to allow for bacterial attachment to food matrices and mimicking naturally contaminated foods. Duplicate samples were used in the experiment for each treatment. Subsequently, each sample was transferred into FILTRA-BAG blender bags (Labplas, Quebec, Canada), homogenized in 225 mL of PALCAM broth (without antibiotics) using a Stomacher 400 Circulator Lab Blender (Seward, UK), and then culture enriched at 37°C for 3–5 h with shaking at 250 rpm.

### Enrichment culture sample preprocessing

Culture-enriched samples were subjected to filtration to remove large food debris. Each sample (250 mL) was transferred into a new FILTRA-BAG blender bag. The liquid sample collected was then passed through the MSFS in series filtration with filters of 250, 180, and 100 µm pore sizes constructed above or alternatively through a BagFilter Pull-up filter bag with porosity of 50 microns (Interscience, St Nom, France). The filtrate was centrifuged at 2,500 × *g* quickly for 20 seconds at room temperature (RT) to further remove food particles. The pellet collected was spread on RAPID’L.mono agar plates (Bio-Rad, Hercules, CA), dried in a biosafety cabinet for 10–15 min, and incubated at 37°C for 24–48 h to determine if *L. monocytogenes* cells were present (blue colonies). The supernatant fraction, presumably containing *L. monocytogenes* cells, was harvested by centrifugation at 10,000 × *g* for 10 min at RT, while the precipitate was analyzed for the presence of *L. monocytogenes* by using the methods described below.

### Culture detection of *L. monocytogenes*

Detection of live *L. monocytogenes* in the pellets obtained from preprocessing of food enrichment cultures was carried out by growing bacterial colonies on *Listeria* selective chromogenic media according to the MFHPB-07 method (14). Briefly, each pellet sample was resuspended in 1–2 mL of phosphate buffered saline (PBS, pH 7.2), divided equally into three aliquots, and spread each on an agar plate containing one of the three solid media: RAPID’L.mono (Bio-Rad), Oxford agar base with Listeria Selective Supplement (MilliporeSigma), and PALCAM base agar with Listeria Selective Supplement (MilliporeSigma). The plates were allowed to dry for 10–15 min, incubated at 37°C for 24–48 h and used to enumerate characteristic colonies of *L. monocytogenes*. On RAPID’L.mono solid media, *L. monocytogenes* produces green-blue colonies (phosphatidyl-inositol phospholipase C (PIPLC) positive) without halos (xylose negative), whereas *L. innocua* appears as white colonies (PIPLC negative) without halos (xylose negative). On Oxford and PALCAM agar media, *L. monocytogenes* forms grayish-green to black colonies due to the hydrolysis of esculin added.

### Magnetic separation of *L. monocytogenes*

Rapid separation of *L. monocytogenes* in the pellets obtained from preprocessing of food enrichment cultures (see above) was performed using a Hyglos *Listeria* capture kit (Hyglos, Bernried, Germany). Briefly, each pellet was resuspended in 2 mL of PBS and dispersed with a cordless pellet pestle (Sigma-Aldrich, Oakville, Ontario) for 5 min. The suspension (approximately 3–3.5 mL) with no visible clumps was split equally, transferred to two 2 mL microcentrifuge tubes each containing one 5 mm stainless steel bead (QIAGEN, Ontario, Canada), and homogenized on a TissueLyser II instrument (QIAGEN, Ontario) at 30 Hz for 2 min. The homogenates were pooled in a 5 mL microcentrifuge tube and mixed with 50 µL of bacteriophage endolysin CWBD-coated magnetic beads (suspension) supplied in the *Listeria* capture kit (Hyglos). After mixing in a circular motion at 1,500 rpm for 15 min in a ThermoMixer device (Eppendorf, Mississauga, Ontario), the suspension was equally divided and transferred to two microcentrifuge tubes. The magnetic beads with bound bacterial cells were collected by magnetic separation (MS) using a DynaMag-2 Magnet magnetic particle concentrator (Life Technologies, Burlington, Canada) with continuous inversion in a rotary mixer for 5 min, washed with PBS containing 0.1% (vol/vol) Tween 20 (PBST) twice, and resuspended in 100 µL PBST. One quarter (25 µL) of the bead suspension was used to detect *L. monocytogenes* by culture plating on RAPID’Lmono agar media, and the remaining sample (75 µL) was used for real-time PCR (RTi-PCR or qPCR) detection of *L. monocytogenes*.

### Extraction of genomic DNA from *L. monocytogenes* bound to magnetic beads

Beads complexed with *L. monocytogenes* in a 75 µL suspension were collected by MS as above, boiled in 50 µL of 50 mM NaOH for 10 min, and then neutralized with 5 µL of 1 M Tris-HCl (pH 8.0) according to the method described (33). The genomic DNA sample thus prepared was used for the amplification of an *L. monocytogenes*-specific DNA target by RTi-PCR.

### RTi-PCR detection of *L. monocytogenes*

RTi-PCR was employed to detect an *L. monocytogenes*-specific DNA target (i.e., the virulence gene *hly*) using a LightCycler 96 system (Roche Diagnostics, Laval, Quebec) and a MicroSEQ *L. monocytogenes* RTi-PCR detection kit (Applied Biosystems, Burlington, Ontario) according to the suppliers’ instructions. The kit is configured to have lyophilized assay beads holding reagents such as DNA polymerase, gene-specific primers and FAM-labeled TaqMan probe, VIC-labeled probe for internal amplification control (IAC), and other necessary components. Preliminary results showed that ≥50 copies of *L. monocytogenes* genomic DNA copies need to be present initially for detecting the target DNA by the kit. To improve the sensitivity of detecting *L. monocytogenes* at a low count in food samples (e.g., 1–5 CFU per 25 g of food) after abbreviated culture enrichment, a single-tube nested RTi-PCR (ST-nRTiP) strategy was implemented by using the kit in conjunction with an additional outer primer set directed to the *hly* gene (GenBank accession number CP020022) of *L. monocytogenes* strain LI0521 (serotype 4b). The primer set (OFP: 5′-CGCAACAAACTGAAGCAAAGGATGCATCTGC-3′; ORP: 5′-GCGGCACATTTGTCACTGCATCTCCGTG-3′), which amplifies an outer 223 bp fragment of *hly* to generate the DNA template for the detection with the RTi-PCR kit, was designed according to the criteria described (34) and synthesized by Sigma (Oakville, Ontario). ST-nRTiP was performed in a total 30 µL reaction volume containing 5 µL of *L. monocytogenes* genomic DNA at various concentrations and 2.5 µL each of the outer primer set (5 µM) using the following cycling parameters: 95°C for 2 min, 30 cycles of two steps (denaturation at 95°C for 15 s and annealing/extension at 68°C for 60 s), and 30 cycles of two steps (denaturation at 95°C for 15 s and annealing/extension at 60°C for 60 s). For comparison, RTi-PCR without nested PCR step was performed using the kit supplier’s recommended cycling parameters: 95°C for 2 min, followed by 40 cycles of two steps (denaturation at 95°C for 10 s and annealing/elongation at 60°C for 30 s). The fluorescence signals acquired from the FAM-labeled TaqMan probe for *hly* and VIC-labeled probe for IAC were analyzed by the Roche LightCycler 96 application software. Samples were classified as positive if the Cq value reached the cut-off value. Unless otherwise stated, all PCR reactions were performed in two technical replicates and two biological replicates.

### Evaluation of a workflow for the detection of *L. monocytogenes* in foods

A workflow was designed and evaluated for the detection of *L. monocytogenes* in foods within a short turnaround time, preferably within a normal workday. The workflow was composed of abbreviated culture enrichment of food samples (3–5 h), preprocessing of enrichment cultures through filtration and centrifugation, magnetic separation of target bacteria, and detection of bacteria by culture plating and a nested RTi-PCR assay. All the processes in the workflow were carried out as described above. Briefly, the evaluation began with the preparation of artificially contaminated ground beef samples containing 1–5 CFU of *L. monocytogenes* in 25 g sample sizes. Each food sample was culture enriched in 225 mL of PALCAM broth (without antibiotics), preprocessed with the MSFS or with a BagFilter Pull-up filter bag, and precipitated through a two-step centrifugation (low and high speed). Each pellet was resuspended in 2 mL of PBS, homogenized using a cordless pellet pestle and a TissueLyser II instrument in the presence of 5 mm stainless steel beads, and subjected to the capture by magnetic separation using the beads coated with bacteriophage endolysin CWBD. Bacteria complexed with beads from each sample were resuspended in a final volume of 100 µL PBST, ¼ and ¾ of which was used for *L. monocytogenes* detection by culture plating on RAPID’L.mono solid media and by a nested RTi-PCR assay described above, respectively. Two independent experiments were conducted to evaluate the workflow.

## RESULTS

### A multi-stage filtration system offers an efficient way of cleaning up food enrichment culture samples

Figure 1 shows an MSFS constructed with an assembly of three series of connected modules. The system was designed such that it can be assembled and disassembled with ease for the replacement of a filter disc. To test the functionality of MSFS, a sample, prepared from 225 mL of culture medium and 25 g of ground beef in a FILTRA-BAG blender bag, was passed through the system that had been equipped with different filter discs of pore sizes 250 (SS mesh), 180 and 100 µm. The system was capable of removing large solid food particulates under a vacuum pump. The liquid filtrate obtained was then applied to a second MSFS system equipped with different filter discs of pore sizes 80, 60, and 40 µm. Multiple repeats of testing the system demonstrated that consecutive filtration through filter discs with a range of pore sizes from 250 to 40 µm effectively eliminated large solid particulates from enriched culture samples and delivered each relatively “clear” preprocessed sample of approximately 225 mL. Centrifugation of filtered samples yielded pellets with wet weights of 3.9 ± 2.2 g (*n* = 10), which presumably contained the target bacteria. Filtration experiments with a commercial BagFilter Pull-up filter bag (50 µm) showed that the bag was as effective as the MSFS as it was able to retain large solid food particulates with wet weights of 14.95 ± 2.53 g (*n* = 16) within the bag. Thus, this commercial bag can be an alternative to the MSFS for preprocessing culture enrichment samples.

### Filtration-based sample preprocessing shortens the time for recovering *L. monocytogenes* from food samples

**Fig 2:**
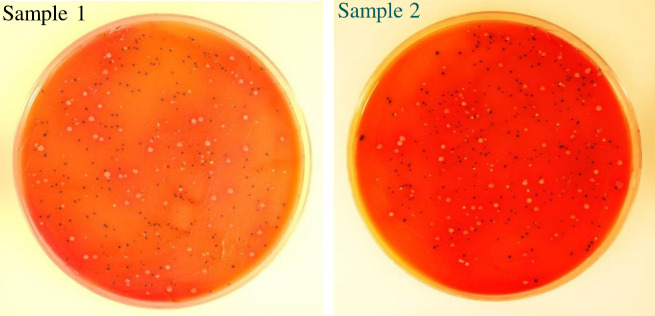
Confirmation by plating of *L. monocytogenes* present in pellets derived from culture enrichment samples. Ground beef samples were each artificially contaminated with 1–5 CFUs of *L. monocytogenes* strain LI0521 (serotype 4b) and culture enriched for 5 h in 225 mL of PALCAM broth (without selective supplements). The pellets, obtained from the preprocessing of enrichment samples using a combination of the MSFS and centrifugation, were resuspended in PBS, plated on RAPID’L.mono agar plates, and incubated at 37°C for 24–48 h. *L. monocytogenes* are indicated by dark blue colonies, while white colonies may represent other *Listeria* or non-*Listeria* spp. The images of colonies were taken after 24 h of incubation at 37°C.

**Fig 3:**
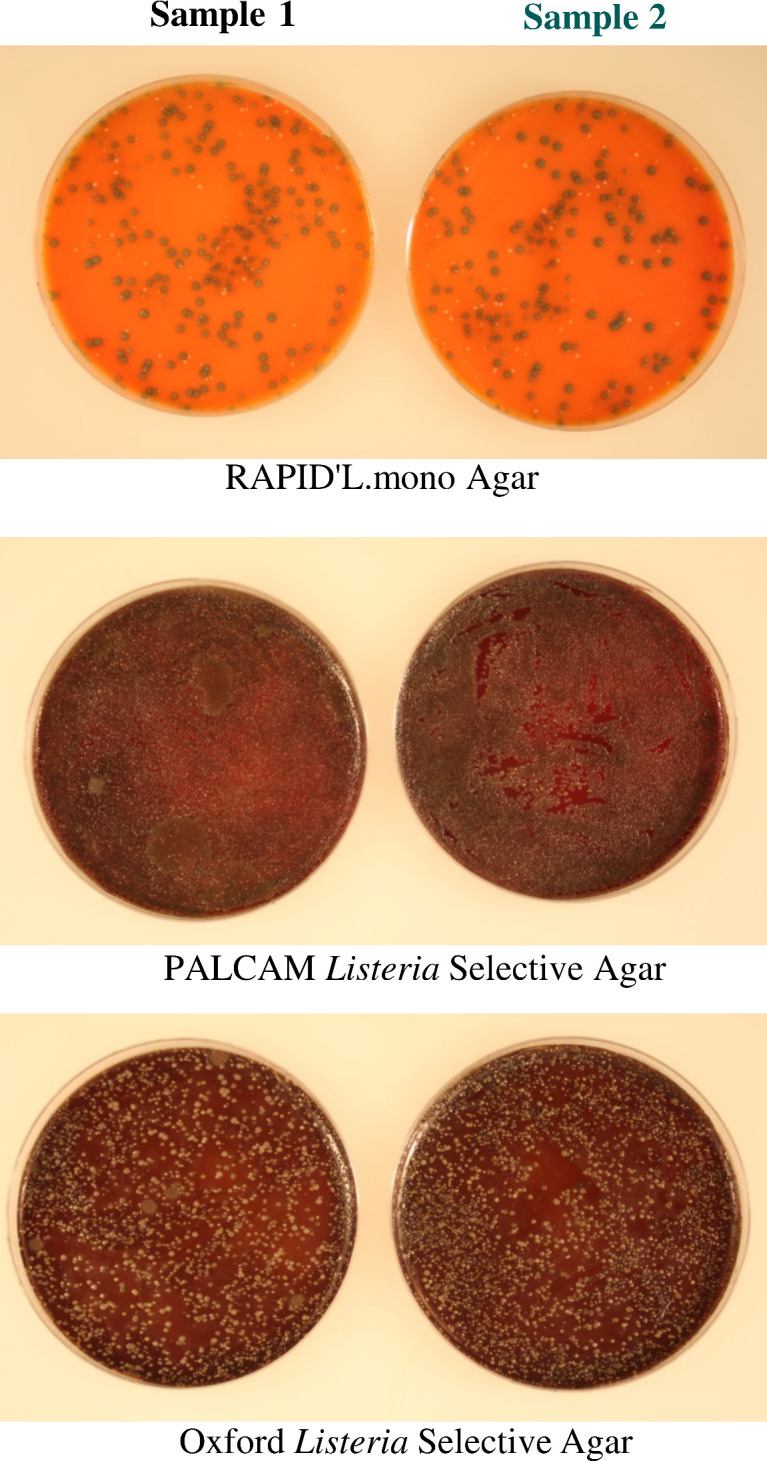
Detection of *L. monocytogenes* in artificially contaminated ground beef samples by culture plating. Ground beef samples (25 g each) were spiked with 1–5 CFUs of *L. monocytogenes* strain LI0521 serotype 4b, culture-enriched in 225 mL of PALCAM broth at 37°C for 3–5 h, and subjected to preprocessing as described in Materials and Methods. The pellets resulting from sample preprocessing were resuspended in PBS and analyzed for *L. monocytogenes* by plating and incubating at 37°C for 24 h on RAPID'L.mono Agar, PALCAM Listeria Selective Agar or Oxford *Listeria* Selective Agar media.

Following culture enrichment of ground beef samples (25 g) each spiked with a low level (1–5 CFU) of *L. monocytogenes* in 225 mL of medium for 3–5 h, the application of a sample preprocessing procedure, i.e., combination of the MSFS and centrifugation, to the enrichment cultures enabled the collection of target bacteria in small pellets. Analysis of the pellets by culture plating revealed the growth of *L. monocytogenes* on RAPID’L.mono agar medium (Fig. 2). Ground beef prior to contamination experiments was found containing no *L. monocytogenes* by culture plating. RAPID’L.mono agar medium was more effective in suppressing the growth of background bacteria in ground beef samples than PALCAM or Oxford Listeria Selective Agar media (Fig. 3). For this study, plating with RAPID’L.mono agar medium is the method of choice for microbiological detection of *L. monocytogenes*.

**Fig 4:**
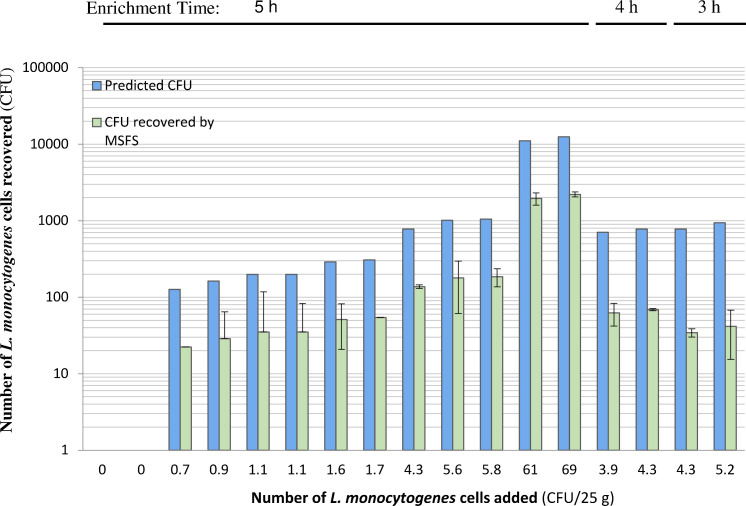
Quantitative recovery of *L. monocytogenes* from artificially contaminated ground beef samples following culture enrichment. Ground beef samples (25 g each) were artificially contaminated with various CFUs of *L. monocytogenes* strain LI0521 serotype 4b, culture-enriched in 225 mL of PALCAM broth at 37°C for 3–5 h, and subjected to preprocessing using a combination of the MSFS and centrifugation. The counts of *L. monocytogenes* were determined by culture plating on RAPID'L.mono Agar media and presented as the average (mean ± SD, *n* = 2, green bar) of two independent experiments each with two replicates. Blue bars represent the counts predicted based on the formula: *N*_*t*_ = *N*_0_ × 2^*n*^ where *N*_*t*_ is the cell number at time *t*, *N*_0_ is the cell number at time *t* = 0, and *n* is the number of generations. The generation time (**G**) for *L. monocytogenes* is taken as 1 h, close to the average generation time of 1.67 h for culturing *L. monocytogenes* from milkshake flavors (35). The number of generation (**N**) is calculated by *n* = *t*/*g*.

**Fig 5:**
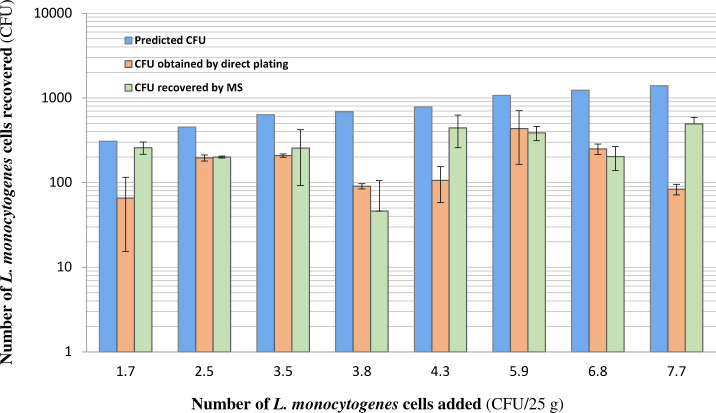
Magnetic separation of *L. monocytogenes* from preprocessed culture enrichment samples. Ground beef samples (25 g each) were artificially contaminated with various numbers (1–8 CFUs) of *L. monocytogenes* strain LI0521 serotype 4b, culture-enriched in 225 mL of PALCAM broth at 37°C for 5 h, and subjected to preprocessing using a combination of BagFilter Pull-up filter bags and centrifugation. *L. monocytogenes* cells were further isolated from preprocessed samples by MS using a Hyglos *Listeria* capture kit, enumerated by culture plating on RAPID'L.mono Agar media (green bar), and compared to the counts determined by culture plating without MS (orange bar). The predicted counts (blue bar) were computed as described in the legend of Fig. 4. Each bar represents the average (mean ± SD, *n* = 2) of two independent experiments each with two replicates.

Culture enrichment represents a time-limiting step for the detection of *L. monocytogenes* in food or environmental samples. Experiments conducted with 25 g ground beef sample units contaminated with a low level of *L. monocytogenes* ranging from 1 to 69 CFU revealed a recovery of ~30 CFUs of target cells from samples contaminated with 1 CFU for a 5 h enrichment or contaminated with 4–5 CFUs for a 3–4 h enrichment (Fig. 4). Recovery of *L. monocytogenes* cells attained 200 CFUs from a 5 h enrichment culture of 4–6 CFUs spiked into ground beef samples. Similar recovery of *L. monocytogenes* from artificially contaminated ground beef was demonstrated using BagFilter Pull-up filter bags (50 µm) to preprocess culture enrichment samples (Fig. 5), in comparison to that obtained with the MSFS. Given that BagFilter Pull-up filter bags performed equally well for sample preprocessing compared to the MSFS and were disposable with ease of operation, they were used in the remaining experiments.

### Magnetic separation specifically isolates *L. monocytogenes* from preprocessed enrichment culture samples

Preprocessed enrichment culture samples, which were obtained by a 5 h culture enrichment of ground beef artificially contaminated with a range of *L. monocytogenes* cell counts (1–8 CFUs) per 25 g sample followed by filtration with BagFilter Pull-up filter bags and centrifugation, were used to isolate the target cells by MS with bacteriophage endolysin-derived CWBD conjugated magnetic beads. Figure 5 showed that magnetic separation of *L. monocytogenes* from preprocessed samples was able to recover the counts of *L. monocytogenes* similar to those obtained by culture plating without MS.

### A commercial RTi-PCR kit coupled with a nested PCR strategy improves the detection sensitivity for *L. monocytogenes*

Assessment of a MicroSEQ *L. monocytogenes* RTi-PCR detection kit with known concentrations of *L. monocytogenes* (10^2^–10^6^ CFUs per PCR reaction) prepared in PBS from pure culture demonstrated an amplification efficiency of 99.1%, which was calculated from a plot of Ct values vs corresponding genomic DNA copy number on a logarithmic scale. The RTi-PCR kit was consistently unable to detect *L. monocytogenes* at a concentration lower than 50 CFUs per PCR reaction, and the limit of detection (LOD) for the assay was 50 CFUs per reaction under the assay condition used (Table 1). Incorporation of an outer primer set directed to the *hly* gene into the kit allowed the implementation of a nested PCR strategy, resulting in the detection of *L. monocytogenes* as low as 12.5 CFUs in a reaction, i.e., a fourfold sensitivity increase (Table 1).

**TABLE 1:** . Limit of detection for the ST-nRTiP assay and the RTi-PCR assay with MicroSEQ *L. monocytogenes* detection kit

Numbers of DNA molecules/reaction		100	50	25	12.5	6.3	3.2	1.6
ST-nRTiP	Signal ratio^*a*^	8/8	8/8	8/8	8/8	7/8	5/8	2/8
	Mean Ct^*b*^	32.30	33.25	34.14	34.74	35.30	35.57	36.68
	SD^*c*^	0.52	0.80	0.79	1.00	0.70	1.18	1.97
MicroSeq *L. monocytogenes* detection kit	Signal ratio	8/8	8/8	7/8	4/8	3/8	2/8	0/8
	Mean Ct	33.80	34.73	35.08	36.32	35.29	36.96	ND^*d*^
	SD	1.24	0.76	1.36	0.64	0.65	0.25	ND^*d*^

^*a*^ Ratio of the positive reactions to total reactions.

^*b*^ The number of cycles required for the fluorescent signal to cross the threshold (background signal).

^*c*^ Negative reactions were excluded from mean and SD calculations.

^*d*^ DNA amplification was not detected.

### Abbreviated culture enrichment integrated with physical separation and real-time PCR techniques accelerates the detection of *L. monocytogenes*

**Fig 6:**
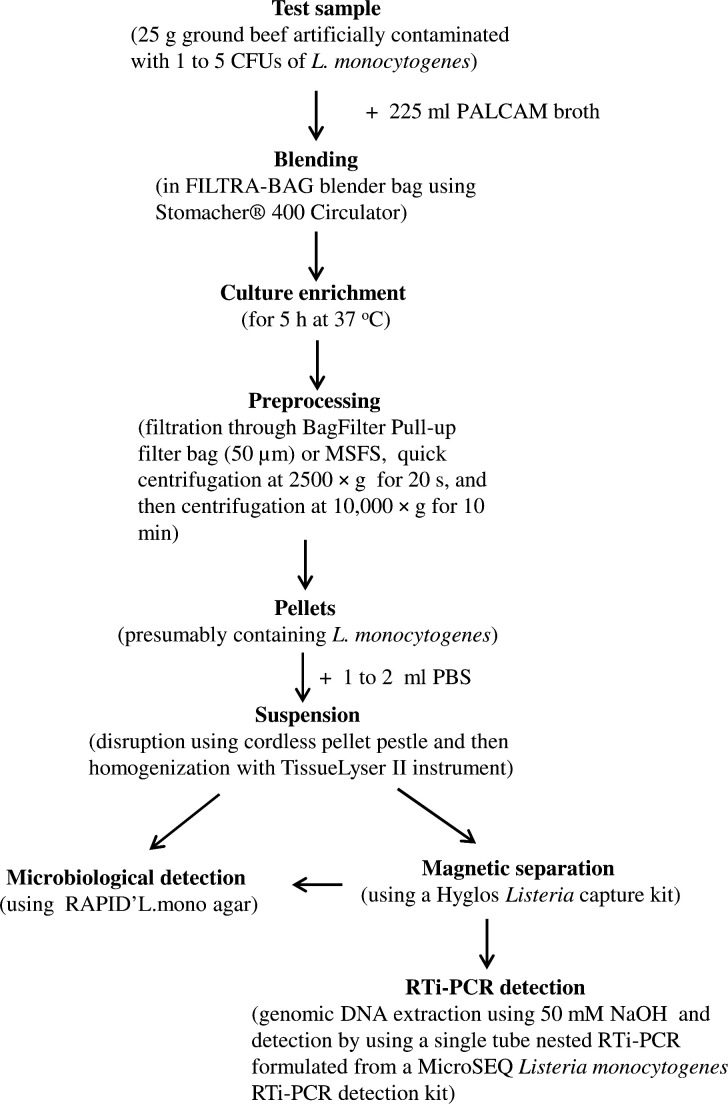
A workflow in an 8 h workday for accelerating the detection of *L. monocytogenes* in artificially contaminated samples. Detailed procedures are described in Materials and Methods. The time taken to complete the tasks is 5 h for culture enrichment, 40 min for culture enrichment sample preprocessing, 30 min for magnetic separation, and 1.5–2 h for detection with RTi-PCR.

Figure 6 shows the design of a workflow to streamline the process for testing *L. monocytogenes* present in artificially contaminated ground beef samples. Evaluation of this workflow with ten 25 g ground beef samples each spiked with a known cell count (1.7–7.7 CFUs) of *L. monocytogenes* demonstrated that the pathogen detection was consistently achieved within a 8 h work day for the bacterial count as low as 2 CFUs in a 25 g sample unit following a 5 h culture enrichment (Table 2).

**TABLE 2:** Rapid detection of *L. monocytogenes* in artificially contaminated ground beef samples using a workflow of a 5 h culture enrichment followed by filtration-based sample preprocessing, MS, and bacterial detection with ST-nRTiP or culture plating

Detection method	CFU per 25 g sample^*a*^							
	7.7	4.3	3.8	3.5	2.5	1.7	−ve	+ve
ST-nRTiP^*b*^	+	+	±	+	+	+	−	+
Culture plating^*c*^	+	+	+	+	+	+	−	+

^*a*^ Twenty-five gram ground beef samples were inoculated with various number of *L. monocytogenes* strain LI0521 serotype 4b; samples for negative control (−ve) and positive control (+ve) were contaminated with 0 and 10^4^ CFU of *L. monocytogenes*, respectively.

^*b*^ Positive (+), negative (−), or inconsistent (−) reactions were observed in two independent experiments.

^*c*^ Culture plating was performed to verify the presence (+) and the absence (−) of typical *L. monocytogenes* colonies in two independent experiments.

## DISCUSSION

The present study demonstrates the design and implementation of a new *L. monocytogenes* detection workflow, which incorporates filtration-based sample preprocessing, magnetic separation, and detection using single-tube RTi-PCR or *Listeria* culture plating technique. This workflow enables the detection of *L. monocytogenes* in a 25 g ground beef sample artificially contaminated with as low as 2 CFUs following a 5 h culture enrichment and permits the detection of this important pathogen in food samples within an 8 h work day. This workflow accelerates the detection of *L. monocytogenes* in food samples, compared to the conventional time-consuming and labor-intensive culture procedure based on selective enrichment and plating for detecting the presence of *L. monocytogenes* in food and environmental samples (30), which involves primary culture enrichment, secondary culture enrichment, and culture plating for a lengthy period of 3–5 days. The workflow makes a significant improvement in the test turnaround time for *L. monocytogenes* detection.

Filtration-based sample preprocessing is an important step in the workflow. Foods naturally contaminated with *L. monocytogenes* typically contain the pathogen at a low level (36), which poses a great challenge to pathogen detection. Thus, culture enrichment through the incubation of a food sample in specially formulated media becomes necessary to increase the number of target bacterial cells but undesirably increases the turnaround time for test results. To minimize this negative impact, the workflow was designed to implement culture enrichment only for 5 h in the present study instead of 24–48 h required for conventional primary and secondary enrichments. Yielding a low concentration of bacteria is expected from a short period of culture enrichment in a large volume of culture broth. Filtration-based sample preprocessing through filter membranes with progressively smaller pore sizes not only allowed the removal of large food particles from enriched samples but also concentrated bacteria to a small pellet that can be resuspended in a desired volume (1–2 mL) of PBS. Filter-based pathogen enrichment technology was demonstrated to hold promise for the detection of *L. monocytogenes*, *Salmonella enterica*, and *Escherichia coli* O157:H7 at around 1 CFU/mL or 1 CFU/g in various food samples (37). An automated cell concentration instrument based on cross-flow microfiltration was developed for rapid concentration and recovery of *S. enterica* cells from aqueous chicken homogenates (38). Despite the noted benefits from filtration-based sample preprocessing, the step has its limitations as it may have also harvested background microflora, which may interfere with the downstream pathogen detection. One obvious solution to the problem is to physically separate from background microflora the bacterial pathogens such as *L. monocytogenes* targeted by the present study.

IMS is a mature technology that has been widely used for the capture of foodborne bacterial pathogens. It often relies on the use of specific antibodies conjugated onto magnetic beads to separate a target microorganism from test samples. For *L. monocytogenes*, both conventional antibodies (24, 26) and endolysin-derived CWBD (27, 28, 39) were explored as a molecular affinity probe to capture the pathogen. Studies of our group and others showed that CWBD-based magnetic separation is more efficient in the recovery of *L. monocytogenes* from samples than antibody-based magnetic separation (27–29). Thus, a magnetic separation step using a bacteriophage endolysin CWBD supplied from a commercial kit was incorporated into the workflow. The separation step was highly effective as it enabled the recovery of nearly all target pathogen cells contained in the preprocessed sample pellets. This step played an important role in the development of an improved workflow for *L. monocytogenes* detection, as it helped to obtain a highly pure sample containing the target pathogen.

The workflow includes the final detection of *L. monocytogenes* present in the samples derived from the magnetic separation. The design of the final detection step involves consideration of the rapid delivery of accurate test results and the need to recover target bacteria from food samples. For such a reason, RTi-PCR was implemented instead of time-consuming conventional biochemical tests in the final step as it was recognized as a rapid, sensitive method for molecular detection of microbial pathogens including *L. monocytogenes* (40, 41). In parallel, the conventional culture plating technique was employed to analyze a portion of an MS-prepared sample. The implementation of an RTi-PCR-based molecular technique was shown to shorten the test result turnaround time, whereas the culture plating method enabled the isolation of the bacterial isolates that can be characterized further by whole-genome sequencing, molecular typing, serotyping, and biochemical tests. Previous studies have reported a number of *L. monocytogenes* genes targeted by RTi-PCR or PCR for pathogen detection, including *hly*, *iap*, *prfA*, and *ssrA* (41–43). In this study, a nested RTi-PCR targeting the *hly* gene was implemented in a single reaction tube for *L. monocytogenes* detection by designing a pair of outer primers used in conjunction with a MicroSEQ *L. monocytogenes* RTi-PCR detection kit (Applied Biosystems). The observed increase in the detection sensitivity was due to the use of a pair of outer primers to boost the concentration of target template DNA for PCR re-amplification. Single-tube nested RTi-PCR has been described for the detection of viral, parasitic, and bacterial pathogens (33, 44, 45). Single-tube nested RTi-PCR is preferable to a traditional nested PCR with two successive rounds of amplification in separate tubes as it eliminates the time for setting up the second round of PCR and thus reduces test turnaround time and the risk of PCR contamination. The nested RTi-PCR is expected to detect all *L. monocytogenes* strains, given that the *hly* gene coding for the virulence factor listeriolysin O is well conserved in and unique to *L. monocytogenes* strains (41, 46). Furthermore, multiple alignment of the *hly* gene homologs from *L. monocytogenes* strains of 12 serotypes (1/2a, 1/2b, 1/2c, 3a, 3b, 3c, 4a, 4b, 4c, 4d, 4e, and 7) revealed annealing sites for the nested RTi-PCR outer primer pair in the conserved regions within the *hly* sequence (data not shown).

The workflow developed in this study provides a unique approach leading to a significant improvement in food testing for contamination with a low level of *L. monocytogenes*. It was capable of reducing the turnaround time significantly down to 8 h on the same day when testing a 25 g sample unit containing as low as 2 CFUs of *L. monocytogenes*; this is in contrast to the next day turnaround time for the detection of *L. monocytogenes* in artificially contaminated food by using a combined culture enrichment and RTi-PCR method (42, 47). Various methods for the detection of *L. monocytogenes* in food have been developed, including culture-based detection, immunological detection, magnetic separation, nucleic acid-based detection, and detection with biosensors (48). In spite of the availability of these methods, possible combinations of these detection methods to form an efficient workflow model remain inadequately investigated. The use of filtration and IMS with Dynabeads anti-*Listeria* beads in combination of RTi-PCR was reported to detect *L. monocytogenes* in artificially contaminated hot-smoked salmon samples with an LOD of 20–40 CFUs/g (49). This study has further explored a combination of several well-characterized methods as a workflow for *L. monocytogenes* detection in food. The workflow was demonstrated to be much more sensitive and efficient for the detection of *L. monocytogenes* in food with an LOD of 2 CFUs/25 g. The improvement was made possible through the implementation of several key steps in the workflow. These steps were (i) recovering and concentrating bacterial cells from entire culture enrichment for a 25 g food sample with a filtration-based sample preprocessing procedure, (ii) separating *L. monocytogenes* from other background bacteria and potential PCR inhibitory materials using a highly efficient magnetic separation based on a bacteriophage endolysin CWBD instead of traditional antibodies, and (iii) detecting the target pathogen with high sensitivity and specificity using a single-tube nested RTi-PCR rather than regular RTi-PCR. This workflow also produced highly pure *L. monocytogenes* isolates after the culture plating step, which is necessary for bacterial isolate characterization using conventional microbiological methods and next-generation sequencing. Testing the workflow using ground beef samples artificially contaminated with one selected *L. monocytogenes* strain may be a limitation of the present investigation, given that *L. monocytogenes* cells in naturally contaminated foods can be damaged by food processing, stress exposure, and storage conditions, leading to an increase in the strain-dependent lag phase duration during culture enrichment (50). Nevertheless, the workflow offers a potentially efficient solution to detect a low level of *L. monocytogenes* from contaminated foods.

In conclusion, the present study has demonstrated the success of designing and testing a workflow model composed of culture enrichment for a short period of 5 h, filtration-based sample preprocessing, magnetic separation, a single-tube nested RTi-PCR, and culture plating for the detection of *L. monocytogenes* in artificially contaminated ground beef samples. The workflow was efficient, sensitive, and capable of providing the sample-to-answer result within 8 h (same day) when testing a 25 g food sample unit contaminated with 2 CFUs of the target pathogen. This workflow has great potential for implementation in food testing laboratories. Future research efforts are needed for the assessment of the described workflow for the detection of various *L. monocytogenes* strains in ground beef and other food matrices, especially those in naturally contaminated foods.
